# Aberrant overexpression of an epithelial marker, 14-3-3σ, in a subset of hematological malignancies

**DOI:** 10.1186/1471-2407-7-217

**Published:** 2007-11-25

**Authors:** Toru Motokura, Yukari Nakamura, Hiroyuki Sato

**Affiliations:** 1 Department of Hematology and Oncology, Faculty of Medicine, The University of Tokyo, Tokyo, Japan

## Abstract

**Background:**

14-3-3σ is a p53-mediated cell-cycle inhibitor in epithelial cells. The expression of 14-3-3σ is frequently altered in cancers of epithelial origin associated with altered DNA methylation. Since its involvement in a non-epithelial tumor is unknown, we examined 14-3-3σ expression in patients with haematological malignancies.

**Methods:**

We analyzed 41 hematopoietic cell lines and 129 patients with a variety of hematological malignancies for 14-3-3σ expression with real-time RT-PCR. We also examined protein levels by Western blot analysis and DNA methylation status of the 14-3-3σ gene by methylation-specific PCR analysis of bisulfite-treated DNA. In addition, mutations of p53 gene were identified by RT-PCR-SSCP analysis and the expression levels of 14-3-3σ were compared with those of other cell-cycle inhibitor genes, CDKN2A and ARF.

**Results:**

The expression levels of 14-3-3σ mRNA in almost all cell lines were low and comparable to those in normal hematopoietic cells except for 2 B-cell lines. On the contrary, 14-3-3σ mRNA was aberrantly overexpressed frequently in mature lymphoid malignancies (30 of 93, 32.3%) and rarely in acute leukemia (3 of 35, 8.6%). 14-3-3σ protein was readily detectable and roughly reflected the mRNA level. In contrast to epithelial tumors, methylation status of the 14-3-3σ gene was not associated with expression in hematological malignancies. Mutations of p53 were identified in 12 patients and associated with lower expression of 14-3-3σ. The expression levels of 14-3-3σ, CDKN2A and ARF were not correlated with but rather reciprocal to one another, suggesting that simultaneous overexpression of any two of them is incompatible with tumor growth.

**Conclusion:**

14-3-3σ, an epithelial cell marker, was overexpressed significantly in a subset of mature lymphoid malignancies. This is the first report of aberrant 14-3-3σ expression in non-epithelial tumors *in vivo*. Since the significance of 14-3-3σ overexpression is unknown even in epithelial tumors such as pancreatic cancers, further analysis of regulation and function of the 14-3-3σ gene in non-epithelial as well as epithelial tumors is warranted.

## Background

14-3-3 proteins regulate many cellular processes such as cell motility, growth, differentiation, apoptosis and cell-cycle checkpoints [1,2] and are involved in tumorigenesis. Among seven members of the 14-3-3 gene family, 14-3-3σ is the most frequently implicated in cancer development [3] and is a mediator of p53 tumor suppressor to arrest cell cycle at the G2 phase by sequestering CDK1/cyclin B complex in cytoplasm [4,5]. 14-3-3σ, also known as stratifin (*SFN*), was identified as an epithelial cell marker protein [6] and was expressed primarily in epidermal epithelia during keratinocyte differentiation. Knockdown of 14-3-3σ expression leads to immortalization of primary human keratinocytes [7]. Furthermore, lack of 14-3-3σ expression associated with DNA methylation of a CpG island residing on the gene is frequently observed in many cancers of epithelial origin such as breast cancers [8,9], liver cancers [10], oral carcinomas [11], and lung cancers [12]. In addition to DNA methylation, estrogen-responsive finger protein, Efp, is implicated in 14-3-3σ down-regulation through proteolysis by its ubiquitin ligase activity [13].

On the contrary, 14-3-3σ can suppress cell death by binding to proapoptotic protein, Bax, and thus could function as an oncoprotein [14]. Consistent with such a possibility, cDNA microarray analysis of pancreatic carcinomas revealed that 14-3-3σ is overexpressed as compared to normal pancreas, which was associated with aberrant hypomethylation in the majority of pancreatic cancers analyzed [15]. 14-3-3σ overexpression was found in papillary but not follicular carcinomas of the thyroid [16]. In addition, increasing 14-3-3σ expression is associated with malignant progression of endometrial carcinoma [17]. These findings raise a 14-3-3σ paradox [1] and suggest that 14-3-3σ is not just a cell-cycle inhibitor or a tumor suppressor but implicated in tumor development as an oncoprotein through anti-apoptotic function or other unknown mechanisms.

Recently, we found that 14-3-3σ was up-regulated in non-epithelial tumor models of rat embryo fibroblasts transformed with c-*myc *and activated H-*ras *[18]. Culture conditions greatly affected the expression levels and methylation status of the 14-3-3σ gene although expression of any other 14-3-3 family member was not altered at all (unpublished data). Sparse culture (*r*-selection) resulted in decreased 14-3-3σ expression along with DNA methylation while the gene was overexpressed and demethylated under a confluent culture condition (*K*-selection) [18]. Therefore, 14-3-3σ may play roles in the development of even non-epithelial tumors either as a tumor suppressor or oncoprotein dependent upon a selection modality governing tumor development of each specific tumor. In addition, non-epithelial astrocytes in the brain were reported to express 14-3-3σ in response to oxidative and DNA-damaging stresses, suggesting a pathological role of 14-3-3σ in non-epithelial cells [19].

Bahtia et al. reported that 14-3-3σ mRNA is expressed at low levels in peripheral blood (PB) lymphocytes [20] and the gene is methylated. In addition, 14-3-3σ protein exists in PB mononuclear cells (MNC) [20,21]. However, the significance of 14-3-3σ expression in non-epithelial, hematological malignancies is unknown. In this study, we examined 129 patients with hematological malignancies for 14-3-3σ expression by real-time RT-PCR and found that aberrant overexpression exists in a subset of patients with mature lymphoid malignancies. This is the first report suggesting the involvement of 14-3-3σ in non-epithelial tumors.

## Methods

### Cell Lines and Clinical Materials

Cell lines and clinical specimens examined in this study were derived from a collection of our laboratory as previously described [22,23]. Clinical specimens were largely from the archived samples obtained for diagnostic purposes and control specimens from healthy volunteers were obtained with written informed consents. The study was approved by the institutional review board. All specimens of hematological malignancies had more than 70% malignant cells as judged with cytosmears.

### Real-Time Reverse Transcription (RT)-Polymerase Chain Reaction (PCR)

RNA extraction and cDNA synthesis were done as described [22]. RNA of each cell line was extracted during log-phase growth. The cDNAs were subjected to real-time PCR for the human 14-3-3σ and GAPDH genes using TaqMan Universal PCR Master Mix (Applied Biosystems, Foster City, CA) according to the manufacturer's protocol with an ABI PRISM 7700 Sequence Detector (Applied Biosystems). Primers and VIC-TAMRA probe for human 14-3-3σ and GAPDH were purchased from Applied Biosystems and used at 0.1 μM and 0.2 μM, respectively. Ratios of the 14-3-3σ to GAPDH values in each sample were standardized by that obtained with Jurkat cells, which was very close to the average value of normal tissues.

### Western Blot Analysis

Cells and clinical specimens were lyzed with 1 × sample buffer (Tris-HCl 60 mM, SDS 2%, dithiothreitol 0.1 M, pH 6.8) and boiled for 5 minutes. Protein concentration was determined by spectrophotometry using BCA Protein Assay Reagent (Pierce, Rockford, IL, USA) [24]. Proteins were separated on a 12% SDS-polyacrylamide gel and subjected to Western blot analysis as described [25]. Quality of protein was judged by Ponceau S staining and cases of degraded proteins were excluded from analysis. Anti-14-3-3σ monoclonal antibody (CS112-2A8 clone; Upstate Biotechnology, Lake Placid, NY) and anti-α-tubulin monoclonal antibody (Ab-1; Oncogene Research Products, San Diego, CA) were used at 1.3 μg/mL and at 0.2 μg/mL, respectively. A secondary antibody was alkaline phosphatase-conjugated rabbit anti-mouse IgG_1 _antibody (Zymed Laboratories) used at 1:1000 dilution.

### Methylation-Specific PCR (MSP) Analysis of Bisulfite-Treated DNA

Genomic DNA was extracted with a conventional method and was modified with sodium bisulfite as described [26]. Sodium bisulfite-treated genomic DNA (100 ng) was subjected to PCR reactions using the following primers: 5'-TGGTAGTTTTTATGAAAGGCGTC-3' and 5'-CCTCTAACCGCCCACCACG-3' for methylated DNA; 5'-ATGGTAGTTTTTATGAAAGGTGTT-3' and 5'-CCCTCTAACCACCCACCACA-3' for unmethylated DNA [8]. These primer sets assessed 4 CpG dinucleotides spanning nucleotides +184–289 (as the first ATG +1, GenBank accession number AF029081) in the CpG island. PCR conditions were as described [20].

### RT-PCR-single-strand conformation polymorphism (SSCP) analysis

The above-mentioned cDNA was subjected to RT-PCR for p53 DNA binding region (exons 4–9, GenBank accession number NM000546). The primers were 5'-CTGTCATCTTCTGTCCCTTC-3' and 5'-TTTCTTTTGCTGGGGAGAGG-3' (0.5 μM each) and the conditions were described previously [27]. After each of two different restriction enzyme treatments (*Hpa*II and *Sau*96I/*Pvu*II), SSCP was done using a non-radioactive method with silver staining as described [28]. When aberrant signals were obtained, amplified DNAs were subjected to direct sequencing using an ABI PRISM BigDye Terminator cycle sequencing Ready Reaction Kit and ABI PRISM Model 310 auto sequencer (Applied Biosystems).

### Statistical Analysis

Mann-Whitney's U test, Kruskal-Wallis test and Scheffe test were used for comparison of 14-3-3σ mRNA levels.

## Results

### Expression of 14-3-3σ mRNA and protein in hematological malignancies

**Figure 1:**
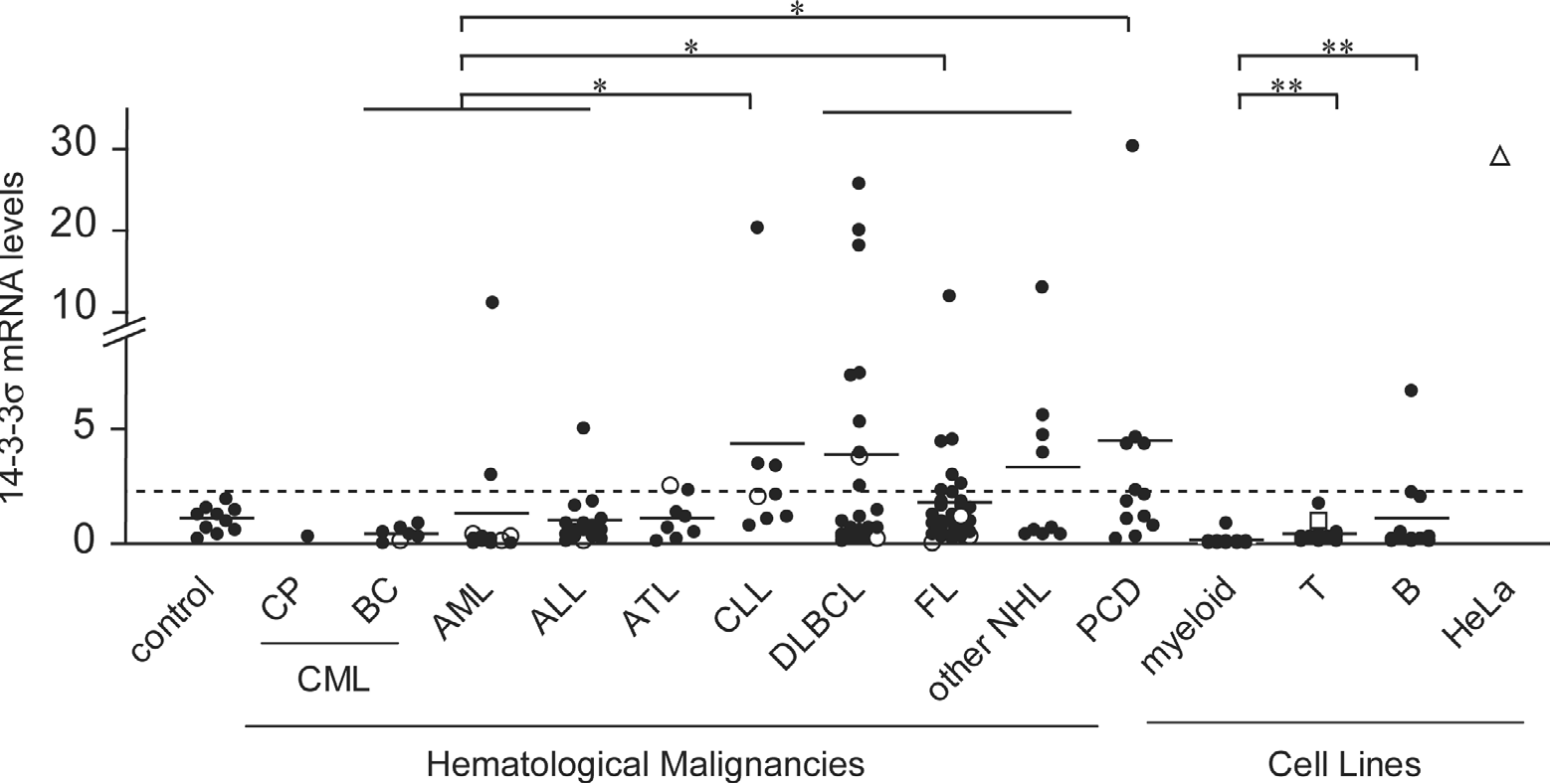
**Expression of 14-3-3σ mRNA in hematological malignancies and cell lines**. Expression levels of 14-3-3σ mRNA were determined by real-time RT-PCR and standardized by that of Jurkat cells (an open square). Dotted line denotes mean plus 2 SDs (2.17) of controls and a mean for each disease or cell line is shown as a horizontal bar. The vertical scales are different between the lower and higher levels. Open circles denote patients with p53 mutations identified by RT-PCR-SSCP analysis. Controls include 5 PBMNC, 3 BMMNC, 2 reactive lymph nodes; CML, 7 patients with blast crisis (BC) and 1 patient with chronic phase (CP) of chronic myeloid leukemia; AML, 12 patients with acute myeloid leukemia; ALL, 16 patients with acute lymphoid leukemia including 3 patients with Burkitt leukemia/lymphoma; ATL, 8 patients with adult T-cell leukemia/lymphoma (1 chronic, 1 acute, and 6 lymphoma variants); CLL, 5 patients with chronic lymphocytic leukemia/small lymphocytic lymphoma and 3 patients with prolymphocytic leukemia; DLBCL, 27 patients with diffuse large B-cell lymphoma; FL, 29 patients with follicular lymphoma; other NHL, 4 patients with T-cell lymphoma, 2 patients with mantle cell lymphoma and 3 patients with unclassified lymphoma; PCD, 12 patients with plasma cell dyscrasias including 1 patient with plasma cell leukemia, 4 patients with Waldenström's macroglobulinemia and 7 patients with multiple myeloma; cell lines include 14 myeloid, 15 T-cell, and 10 B-cell lines (all listed in reference [23]) and an epithelial cell line, HeLa (an open triangle). Asterisks denote statistical significance by Scheffe test after combining CML BC, AML and ALL as a group of acute leukemia and DLBCL, FL and other NHL as a group of NHL: * P < 0.05, ** P < 0.01.

We examined 41 hematopoietic cell lines and clinical specimens of 129 patients with a variety of hematological malignancies for expression of the 14-3-3σ gene with real-time RT-PCR. The expression in normal tissues such as bone marrow (BM) and PB MNC and reactive lymph nodes were also examined as controls. The expression levels in almost all cell lines examined were detectable but low and comparable to those in normal tissues. Only in two B-cell lines, i.e., BALL-1 and LBW2, the expression levels of 14-3-3σ mRNA increased beyond the mean plus 2 standard deviations (SDs) of the controls (mean 1.03, SD 0.57) as shown in Figure 1. Myeloid cell lines expressed significantly less than lymphoid cell lines (P < 0.0001 by Kruskall-Wallis test). On the contrary, the expression levels were frequently elevated among the clinical specimens of patients with a variety of hematological malignancies and significantly different among the types of diseases (P = 0.001 by Kruskall-Wallis test). In patients with mature lymphoid malignancies, increased expression was found in 30 (32.3%) of 93 patients (chronic lymphocytic leukemia 3/8, diffuse large B-cell lymphoma 9/27, follicular lymphoma 7/29, adult T-cell leukemia/lymphoma 2/8, other lymphomas 4/9, plasma cell dyscrasia 5/12) whereas only 3 (8.6%) of 35 patients with acute leukemia expressed 14-3-3σ at the increased levels (acute lymphoid leukemia 1/16, acute myeloid leukemia 2/12, blast crisis of chronic myeloid leukemia 0/7).

**Figure 2:**
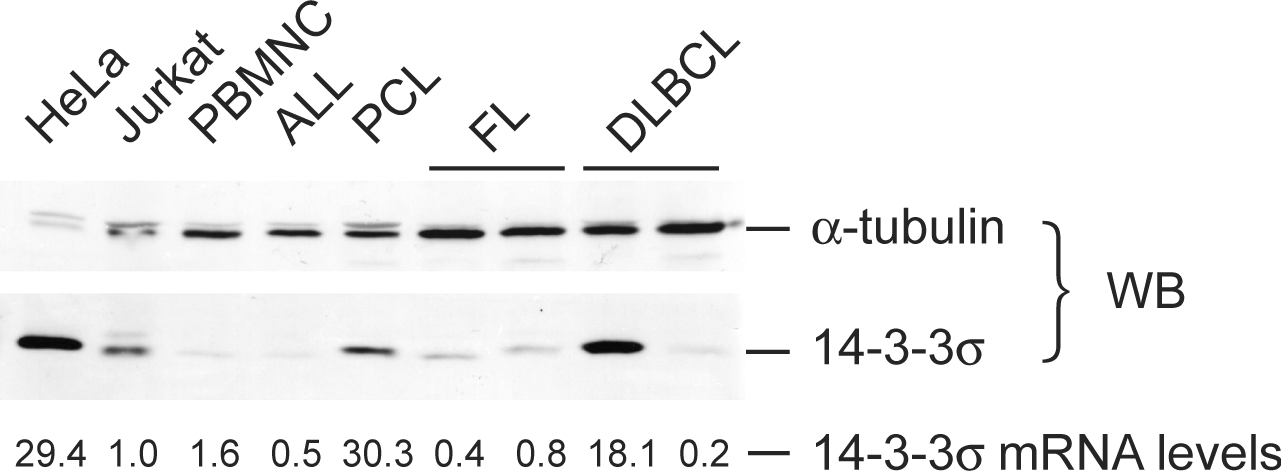
**14-3-3σ protein expression in hematological malignancies**. Western blot analysis of whole lysates (20 μg/lane) of clinical specimens of hematological malignancies was done with anti-14-3-3σ monoclonal antibody. A representative result is shown with HeLa cells (2 μg/lane) as a control. Anti-α-tubulin antibody was used to show the protein loading. 14-3-3σ mRNA expression levels determined by real-time RT-PCR and standardized to that of Jurkat cells are shown below.

Since 14-3-3σ expression is regulated post-translationally, we also examined protein levels in 34 patients with materials available enough to Western blot analysis. The 14-3-3σ protein is readily detectable and roughly reflected the mRNA levels (Figure 2). In patients with high mRNA expression levels comparable to that of a HeLa epithelial cell line, the protein levels in hematological malignancies were well below that of HeLa (see one tenth amount of lysate was loaded for HeLa cells), suggesting the presence of different regulatory mechanisms between epithelial and non-epithelial cells.

### DNA methylation status of the 14-3-3σ gene is not associated with its expression

**Figure 3:**
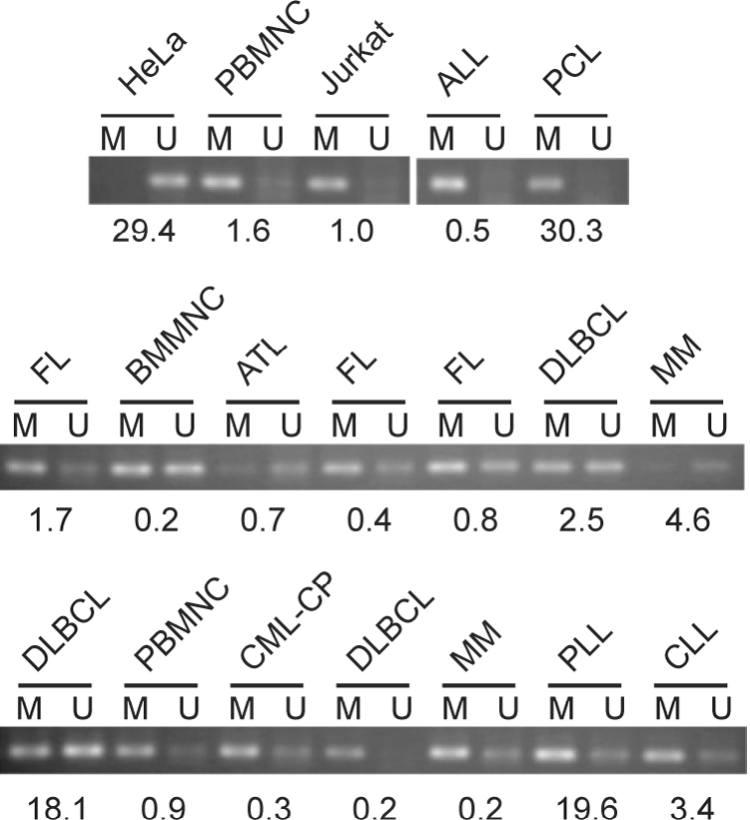
**MSP analysis of DNA methylation status of the 14-3-3σ gene**. Bisulfite-treated genomic DNA was subjected to MSP and amplified products were separated on 1.5% agarose gel. Representative results are shown. The 14-3-3σ mRNA expression levels determined by real-time RT-PCR are shown below. M and U denotes methylated and unmethylated alleles, respectively. PCL denotes plasma cell leukemia; MM, multiple myeloma; CML-CP, chronic phase of chronic myeloid leukemia; PLL, prolymphocytic leukemia.

Since the expression levels and DNA methylation status of the 14-3-3σ gene is highly related in epithelial tumors, we examined the methylation status in 21 patients with MSP analysis of bisulfite-treated DNA. Representative results are shown in Figure 3. In PBMNC and BMMNC with low expression of 14-3-3σ mRNA, DNA was not always methylated and similar results were obtained in patients with malignancies. A limited number of patients showed unmethylated status and did not necessarily express high levels of 14-3-3σ mRNA and vice versa. These results suggest that expression of 14-3-3σ in hematological malignancies is not mainly affected by the DNA methylation status to the extent observed in epithelial tumors. However, further study with a larger series of patients is required to confirm the finding.

### Comparison with CDKN2A and ARF expression and impact of p53 mutations

**Figure 4:**
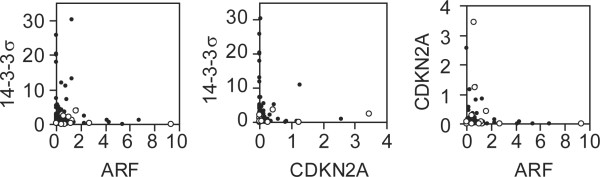
**Comparison of mRNA expression levels between three cell-cycle inhibitors, 14-3-3σ, ARF and CDKN2A**. ARF and CDKN2A mRNA levels were determined by semi-quantitative RT-PCR previously [23]. For each pair of cell-cycle inhibitors, expression levels are plotted in scattered plots. Open circles denote patients with p53 mutations identified by RT-PCR-SSCP analysis. Correlation coefficients for p53 wild-type and mutated patients are: 14-3-3σ vs. ARF, -0.074 and -0.129; 14-3-3σ vs. CDKN2A, -0.033 and -0.391; ARF vs. CDKN2A, -0.044 and -0.177, respectively.

14-3-3σ is an effector molecule downstream of p53 tumor suppressor [4] whereas ARF protein derived from the CDKN2A/ARF locus functions upstream of p53 [29]. ARF expression activates p53 and thus can arrest the cell cycle at the G1 and G2 phases through direct activation of p21^CIP1 ^and 14-3-3σ, respectively [3,30]. In our previous study, we had examined the same collection of clinical specimens for expression of CDKN2A and ARF mRNAs with β-actin as a control [23] and then we compared the expression levels of these mRNAs. As shown in Figure 4, high expression of ARF mRNA was not accompanied by its downstream event, i.e., induced expression of 14-3-3σ mRNA. Because of possible presence of p53 mutations disrupting the signaling pathway from ARF to 14-3-3σ, we examined these patients with RT-PCR-SSCP analysis to identify patients with p53 mutations. Twelve mutated patients were found out of 127 patients examined and distributed in a variety of malignancies as shown in Figure 1 (see open circles). The mutated patients expressed significantly higher levels of ARF mRNA and lower levels of 14-3-3σ than non-mutated ones (P < 0.05 in either case by Mann-Whitney's U test, Figure 4). Thus, p53 mutation appears to disrupt the signaling pathway from ARF to 14-3-3σ. In normal keratinocytes, 14-3-3σ expression is accompanied by CDKN2A expression but in hematological malignancies 14-3-3σ expression was rarely accompanied by CDKN2A expression. Interestingly, CDKN2A and ARF expression levels were not correlated either but rather reciprocal.

## Discussion

The 14-3-3σ gene is highly expressed in epithelial cells and implicated in the development of many cancers of epithelial origin. In the present study, 14-3-3σ expression was found at low levels in normal PB and BM MNC and lymph nodes, consistent with the previous observation by Bahtia et al [20]. We also found that the expression of 14-3-3σ mRNA and protein increased in a substantial number of patients with hematological malignancies. The clinical specimens examined in this study were hardly contaminated with 14-3-3σ-overexpressing epithelial cells. There remains a possibility of normal cells admixed in tumor specimens may upregulate 14-3-3σ expression although expression by small number of normal cells should hardly explain the cases of high expression comparable to HeLa cells. The increased expression was not confined to any specific disease but found in a category of mature lymphoid malignancies. These findings suggest that the aberrant expression is implicated in the development of non-epithelial tumors as well as epithelial cancers. However, there remains to investigate whether the increased expression may reflect normal development of lymphoid cells.

The 14-3-3σ mRNA levels in almost all cell lines were suppressed whereas those in clinical specimens were highly variable. Our previous study revealed that *r*-selection favored cells with low expression of 14-3-3σ and thus, the establishment of cell lines may also select clones which express 14-3-3σ at low levels rather than at high levels. The bias of cell line establishment has also been debated regarding other aberrations such as CDKN2A/ARF locus deletion [31]. During the development of acute leukemia, *r*-selection may well be active since leukemia cells can grow rapidly in general but not localized bone marrow. It is consistent with the finding that upregulation of 14-3-3σ was rare in acute leukemia. On the other hand, high-cell-density selection (*K*-selection) was associated with 14-3-3σ overexpression and emergence of multidrug resistance [18,32]. Furthermore, 14-3-3σ expression contributes to drug resistance and loss of 14-3-3σ expression sensitizes cancer cells to anticancer agents [3,33]. The concepts of *r*- and *K*-selection did not constitute a true dichotomy but merely represent endpoints of a spectrum. Selection modalities are relative and determined by the interaction between tumor cells and their microenvironment. Therefore, *K*-selection may work during the development of tumors *in vivo *especially tumor-forming malignancies and 14-3-3σ overexpression in a subset of patients with mature lymphoid tumors may reflect the selection processes that were more *K *than those in other patients. However, its prognostic significance remains to be investigated.

In many epithelial tumors, DNA methylation status is highly associated with the expression levels of 14-3-3σ. However, the present observation suggested that unknown mechanisms other than DNA methylation are involved in the aberrant expression in hematological malignancies. In addition, the protein level in HeLa cells was much higher than those in hematological malignancies even in patients whose mRNA levels were comparable to that of HeLa cells. The comparison between three cell-cycle inhibitors, 14-3-3σ, ARF, and CDKN2A, suggested that their associated expressions found in normal epithelial cells were lacking. The disrupted link between ARF and 14-3-3σ may well be explained at least in part by the presence of p53 mutations. However, these comparisons were made between data obtained by different methods with a small number of p53-mutated cases. Therefore, confirmation with a larger series of patients is required. Intriguingly, expression levels of any two of them were all reciprocal. Simultaneous overexpression of any two of them seem to be incompatible with tumor development and overexpression of any one of them may indicate that some parts in the braking systems of these cell-cycle inhibitors are broken [34]. However, the regulatory mechanisms and function of 14-3-3σ in non-epithelial cells remain unknown. Even in epithelial tumors, the function of 14-3-3σ overexpressed in pancreatic cancers is enigmatic [35]. The regulation and function of the 14-3-3σ gene in non-epithelial lymphoid malignancies require further analysis.

## Conclusion

We found that 14-3-3σ, an epithelial cell marker, was overexpressed significantly in a subset of mature lymphoid malignancies *in vivo*. The expression levels of 14-3-3σ mRNA were not associated with the DNA methylation status of the CpG island. These findings warrant further analysis of regulation and function of the 14-3-3σ gene even in non-epithelial tumors such as hematological malignancies.

## Competing interests

The author(s) declare that they have no competing interests.

## Authors' contributions

TM designed the study, carried out the experiments, analyzed and interpreted data, and wrote the manuscript. YN carried out RT-PCR-SSCP analysis and HS carried out real-time RT-PCR analysis. All authors read and approved the final manuscript.

## Pre-publication history

The pre-publication history for this paper can be accessed here:


